# Scaling up orphan crop research: genebank genetics highlight geographic structure in cultivated cowpea from 10 617 global accessions

**DOI:** 10.1111/tpj.70777

**Published:** 2026-03-14

**Authors:** Sofie Pearson, Adrian Hathorn, Shichao Sun, Alan Cruickshank, Tracey Shatte, Paulino Munisse, Mercy Macharia Wairimu, Joann Conner, Anna Koltunow, Jean‐Philippe Vielle‐Calzada, Peggy Ozias‐Akins, Takayoshi Ishii, Matteo Dell'Acqua, Sally Norton, Yongfu Tao, David Jordan, Emma Mace

**Affiliations:** ^1^ Queensland Alliance for Agriculture and Food Innovation (QAAFI) The University of Queensland, Hermitage Research Facility Warwick Queensland 4370 Australia; ^2^ Shenzhen Branch, Guangdong Laboratory of Lingnan Modern Agriculture, Agricultural Genomics Institute at Shenzhen Chinese Academy of Agricultural Sciences Shenzhen 518120 China; ^3^ Queensland Department of Primary Industries (DPI) Hermitage Research Facility Warwick Queensland 4370 Australia; ^4^ Mozambique Institute of Agricultural Research Maputo Mozambique; ^5^ Institute of Plant Sciences Scuola Superiore Sant'Anna Pisa Italy; ^6^ Department of Horticulture and Institute of Plant Breeding, Genetics and Genomics University of Georgia Tifton Georgia 31793 USA; ^7^ Queensland Alliance for Agriculture and Food Innovation (QAAFI) The University of Queensland, St Lucia Brisbane Queensland 4072 Australia; ^8^ Grupo de Desarrollo Reproductivo y Apomixis Unidad de Genómica Avanzada Laboratorio Nacional de Genómica Para la Biodiversidad, Cinvestav Irapuato Guanajuato CP36825 México; ^9^ International Platform for Dryland Research and Education (IPDRE) Tottori University Tottori 680‐0001 Japan; ^10^ Arid Land Research Center (ALRC) Tottori University Tottori 680‐0001 Japan; ^11^ Australian Grains Genebank Horsham Victoria Australia

**Keywords:** genetic structure, legume breeding, duplicates, geographic structure, biogeography, orphan crop, opportunity crop, germplasm, pulse, phylogenetics

## Abstract

*Vigna unguiculata* (L.) Walp. is a dryland legume crop, providing essential food and nutritional security for millions of people across the semi‐arid tropics, in Africa, Asia and Latin America. However, as a typical ‘orphan crop’, cowpea has long remained underrepresented in global genomic research to support crop improvement. Here, we conducted the largest genetic diversity analysis of cowpea to date, comprising 10 617 accessions sourced from seven international collections. Using genotyping‐by‐sequencing, we characterised the global patterns of genetic diversity, assessed redundancy within and across collections, and examined the geographic structure of the cowpea global allele pool. Our results revealed nine distinct genetic groups with clear geographic associations and fine‐scale population differentiation, reflecting dispersal history, regional adaptation and the influence of modern breeding. Duplication across collections was detected, highlighting the need for improved curation and integration of germplasm resources. Landraces from sub‐Saharan Africa do not fully capture the genetic diversity present in several other geographic regions, indicating the existence of abundant and untapped genetic resources worldwide. These findings not only provide insights into the genetic structure and evolutionary history of cowpea but also offer a valuable foundation for harnessing global germplasm diversity to enhance breeding potential and accelerate crop improvement.

## Significance Statement

Cowpea genetic diversity assessments have focused on African germplasm with limited exploration of cowpea germplasm from multiple genebanks and origins. This cowpea collection revealed that cowpea's dispersal history throughout the globe is synonymous with its genetic structure, highlighting opportunities to enhance crop improvement by utilising untapped worldwide genetic resources.

## INTRODUCTION

Global food security remains a pressing issue, with an estimated 1 billion people currently underfed, a situation that is likely to intensify as the global population is predicted to surpass 9 billion by 2050 (Gu et al., 2021). Meeting this future demand will require increasing food production while meeting sustainability objectives (Tilman et al., 2011; Van Dijk et al., 2021). However, relying solely on improvements in major staple crops is unlikely to meet this target (Ray et al., 2013). Diversifying food sources is therefore imperative to ensure nutritional security (Gruber, 2017). Cultivating orphan crops (those which lack advanced scientific research and breeding) offers a promising avenue for diversification and sustainability. Orphan crops include cereals, legumes and root crops often cultivated in limited areas in Africa, Asia and Latin America, play an important cultural role and are vital for the nutrition and livelihood of many households (Ye & Fan, 2021). These crops possess desirable traits such as resilience to extreme climatic conditions and high nutritional value (Tadele, 2019) and are also referred to as ‘opportunity crops’ (Yang et al., 2025). Additionally, plant genetic resources including landraces and wild crop relatives conserved in international genebanks are a valuable reservoir of genetic diversity, offering access to novel allelic variation that could contribute to sustainable intensification of yields, providing resistance to diseases and abiotic stress, and increasing nutritional quality (Ye & Fan, 2021).

Genebanks play a critical role in safeguarding plant genetic resources by storing seeds from diverse species, including orphan crops. These large diverse collections promote conservation and support plant breeding and crop improvement, especially in the face of changing environmental conditions (Asdal & Guarino, 2018). This includes maintaining collections of crop seeds, including traditional and heirloom varieties that may possess traits such as pest resistance, drought tolerance and adaptability to low‐input environments (Hay & Probert, 2013). Effective management of these genetic reservoirs is crucial for developing cultivars capable of addressing emerging agricultural challenges (Tanksley & Mccouch, 1997). Therefore, accurate identification and conservation of genetic diversity within collections are essential for optimising breeding programmes (Mascher et al., 2019). Previous studies assessing diversity in genebanks include major staple crops such as wheat (Schulthess et al., 2022), barley (Milner et al., 2019) and rice (Mccouch et al., 2012), but limited studies have assessed the diversity in orphan crops.

Cultivated cowpea is a globally important grain legume, cultivated on approximately 15 million hectares, producing on average 9.7 million tonnes in the past 5 years (FAO, 2025) and provides a major source of nutrition and income for hundreds of millions of households in the semi‐arid tropics, including Africa, Asia and Latin America (Boukar et al., 2019; Dixit & Satheesh Naik, 2025). Despite this importance, cowpea has historically received comparatively limited breeding research investment and genomic resource development relative to major legumes such as soybean, common bean and chickpea. This genomic underrepresentation has led to its classification as an ‘orphan crop’. However, cowpea now occupies a transitional zone, where research is still underfunded relative to its significance but advances in international breeding efforts (Kim et al., 2025) and genomic resources (Liang et al., 2024; Lonardi et al., 2019) are increasingly supported. As genomic resources expand, well‐characterised genebank collections remain essential for accessing the genetic diversity needed to drive further improvement in cowpea. The largest genebank collections of *Vigna unguiculata* (L.) Walp include the International Institute of Tropical Agriculture (IITA) in Nigeria, the United States Department of Agriculture (USDA) National Plant Germplasm System in the USA and the National Bureau of Plant Genetic Resources (NBPGR) in India. Cultivated cowpea are grouped under *V. unguiculata* subsp. *unguiculata*, which is subdivided into five cultivar groups (c.g.) based on seed and pod traits: (1) *Unguiculata* (cowpea or black‐eyed bean, widely cultivated as a pulse or vegetable with more than 16 ovules per pod); (2) *Biflora/Cylindrica* (catjang, common in India, primarily used for forage and has short erect pods with less than 17 ovules per pod and smooth seed); (3) *Sesquipedalis* (yardlong or asparagus bean, common in Asia, grown as a vegetable with very long pods; also known as *Vigna unguiculata* subsp. *sesquipedalis* (L.) Verdc.); (4) *Textilis* (a rare form once cultivated for fibre in Africa with long floral peduncles); and (5) *Melanophthalmus* (grown mostly in the Americas, has less than 17 ovules per pod and includes blackeye pea types) (Pasquet, 2000). Cowpea seeds, leaves and green pods are all utilised and provide essential micronutrients such as zinc and iron, which are often lacking in other legumes (Abebe & Alemayehu, 2022; Jayathilake et al., 2018). Comprehensive characterisation of genebank collections can increase improvement in cultivar development, trait discovery and translate genomic advancements into practical breeding outcomes.

In sub‐Saharan Africa, smallholder farmers are the main producers and consumers of cowpea (Bolarinwa et al., 2022) and often grow it as an intercrop with cereals and other crops (Ongom et al., 2023). Cowpea compensates for the nitrogen loss by cereals through sustainable nitrogen fixation in farming systems (Kussie et al., 2024). Cowpea's centre of domestication is in Africa; from there it has spread to all continents and exhibits high resilience to drought environments and poor soil conditions. However, yields are often adversely affected by cowpea's susceptibility to pests (such as aphids, weevils and pod borers) and diseases (such as Anthracnose and fusarium wilt) (Singh & Allen, 1979). These yield challenges pose a threat to global food security, highlighting the need for continuous breeding efforts. Characterising global germplasm is critical to explore the untapped resource to meet the challenges in cowpea breeding.

Over the last few decades, significant progress has been made in characterising the genetic diversity of cowpea. Genetic studies have consistently highlighted West Africa as the primary centre of cowpea diversity, with substantial variation also found across Southern and Eastern Africa, Asia and the Americas (Fang et al., 2007; Fiscus et al., 2024; Huynh et al., 2013; Sodedji et al., 2021; Xiong et al., 2016). Multiple studies have focused on the diversity in specific geographic regions, including Western Africa (Egbadzor et al., 2014, Gbedevi et al., 2021), Southern Africa (Chipeta et al., 2025; Macharia et al., 2025; Nkhoma et al., 2020), Eastern Africa (Desalegne et al., 2016; Ketema et al., 2020), the Iberian Peninsula (Carvalho et al., 2017), China (Chen et al., 2017) and Korea (Seo et al., 2020). Alongside these diversity studies, cowpea breeding programmes have been established across West, Central, Eastern and Southern Africa, as well as in Brazil, the USA and parts of Asia (Singh et al., 1997). These programmes typically draw upon regionally adapted landraces and elite lines to target key agronomic constraints such as drought and heat stress, insect pests, early maturity, grain quality and dual‐purpose grain‐fodder performance (Boukar et al., 2016, 2020). Because these breeding priorities vary across environments, they strongly shape germplasm collection strategies; for example, breeding efforts focused on low‐input resilience source germplasm from arid or marginal production regions. However, effective breeding requires a clear understanding of the breadth and structure of available genetic diversity, as this underpins the ability to identify novel alleles, incorporate underutilised variation, and assess the comparative value of material used across programmes (Boukar et al., 2019; Smith et al., 2015). Despite regional progress, the relationship among cowpea accessions from a comprehensive global geographic distribution remains poorly resolved. Although global core collections have been developed (Fatokun et al., 2018; Mahalakshmi et al., 2007; Muñoz‐Amatriaín et al., 2021), most rely heavily on traditional cultivars from West Africa with limited representation from outside the continent, leaving gaps in understanding the full extent of global cowpea diversity. Integrating larger and more geographically diverse collections is therefore essential to capture the untapped genetic variation required to support breeding programmes worldwide and to position cowpea as a valuable genetic resource for global food and nutritional security.

Here, we explore a curated selection of the global gene pool of cowpea to understand patterns of genetic diversity and population structure. The collection is particularly valuable because it integrates accessions from five genebanks and two collections, capturing a broad spectrum of geographic origins and genetic backgrounds. We assembled a dataset of 10 617 accessions, approximately 23% of the total global cowpea accessions, representing both core and diverse germplasm from six continents and 121 countries, and applied cost‐effective and robust reduced‐representation sequencing to generate high‐quality genotypic data. The main objectives of this study were to (1) characterise a global collection of cowpea from seven international collections, (2) identify identical accessions within and between collections using genotypic data, and (3) assess the composition of the global cowpea collection in the context of diversity and genetic structure.

## RESULTS

### Metadata‐based summary of the global cowpea collection (taxonomy and improvement status)

A total of 10 617 cultivated cowpea (*V. unguiculata*) accessions and one sister subspecies accession (*Vigna unguiculata* subsp. *stenophylla*) were used in this study and assessed with 4290 SNP markers (Figure S1). These accessions originated from diverse geographic regions and seven collections. The majority of these accessions were from the USDA Genebank (63.4%; *n* = 6735), followed by the IITA Genebank, Nigeria (18.3%; *n* = 1942), representing the core collection of cowpea accessions described by Mahalakshmi et al. (2007). The Australian Grains Genebank (AGG), Australia (7.4%; *n* = 791); the National Agriculture and Food Research Organization (NARO) Genebank, Japan (3.9%; *n* = 414); a collection from the University of California, Riverside (UCR), USA (3.3%; *n* = 351) representing the mini‐core collection described by Muñoz‐Amatriaín et al. (2021); a collection from the National Genebank of Mozambique at the Instituto de Investigação Agrária de Moçambique (IIAM; 2.6%; *n* = 271); and a Central and Southern American collection from Unidad de Genómica Avanzada, Langebio Cinvestav, Mexico (LAN; 1.1%; *n* = 114) also constituted the global collection.

**Figure 1:**
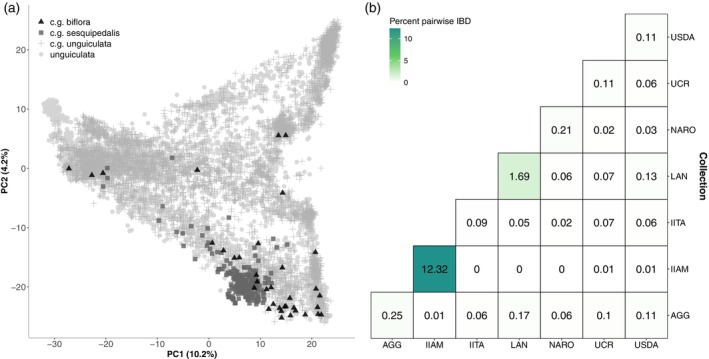
Genetic diversity of the cultivated global cowpea collection. (a) Principal component (PC) analysis of 10 617 accessions colour‐coded based on cultivar group (c.g.), where black triangles = biflora (*n* = 36), grey squares = sesquipedalis (*n* = 240), grey plus = unguiculata (*n* = 5752) and grey circles = species level *Vigna unguiculata* or subspecies *unguiculata* combined (*n* = 4589). (b) Per cent pairwise identity by descent (IBD) greater than zero between collections. The percentage is calculated from the total number of pairs between each comparison compared with observed pairwise IBD values greater than zero.

The majority of cultivated cowpea assessed in this study belonged to the *unguiculata* cultivar group (*n* = 5752), with smaller representations from the *sesquipedalis* (*n* = 240) and *biflora* (*n* = 36) groups (Figure 1a). Forty‐three per cent of the accessions had passport data at the *unguiculata* species (*n* = 3670) or subspecies (*n* = 919) level (Table S1). Cultivated cowpea accessions were classified into six improvement categories: Advanced/Improved Cultivar, Breeding Line, Landrace/Traditional Cultivar, Weedy, Wild and Unknown (Figure S2). The AGG collection consists primarily of Breeding Lines (74%), with a small percentage of Landrace/Traditional Cultivars (15%) and Unknown accessions (11%). In contrast, the IITA, IIAM, NARO and UCR collections contain a large proportion of Landrace/Traditional Cultivars (86, 100, 79 and 74%, respectively). IITA and UCR also contain Breeding Lines (10 and 22%, respectively), while NARO also contains Unknown (12%) and Weedy (6%) accessions. The LAN collection contains mostly Unknown (98%) and Advanced/Improved Cultivars (2%). The USDA Genebank exhibited greater diversity, including Landraces (42%), Unknown (35%), Advanced/Improved Cultivars (19%) and Wild accessions (3%) (Table S2).

### Identification of breeding material from genomic signatures

**Figure 2:**
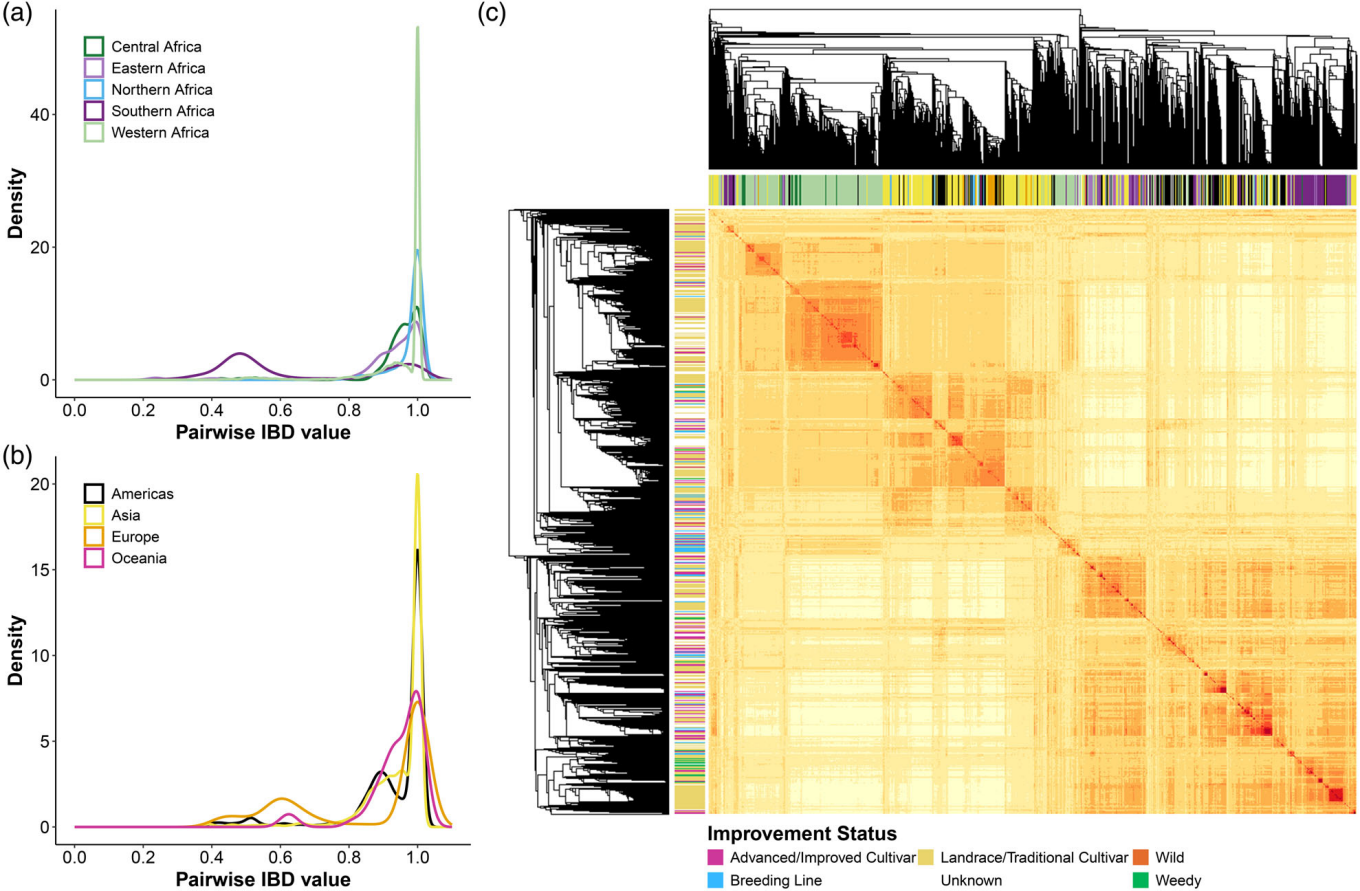
Genetic relatedness of the cultivated global cowpea collection. (a, b) Pairwise identity by descent (IBD) frequency distribution for nine geographic regions. (c) Genomic relationship matrix of 10 617 accessions, where yellow = dissimilar and red = similar. Accessions are coloured based on their geographic origin in the columns (colour key in [a] and [b]) and improvement status in the rows (colour key presented below the heatmap).

Pairwise identity by descent (IBD) was calculated for all 10 617 cultivated cowpea and assessed for differences between collections and geographic provenance. Pairwise IBD values ranged from 0 to 0.17% between collections and from 0.09 to 12.3% within collections (Figure 1b). Higher pairwise IBD was observed between AGG‐LAN (0.17%), LAN‐USDA (0.13%) and AGG‐USDA (0.11%), while no pairwise IBD was detected between accessions IIAM‐LAN, IIAM‐IITA and IIAM‐NARO. The highest pairwise IBD was found within the IIAM Genebank (12.3%), while all other collections had values below 2%. All geographic regions have an IBD peak around 0.95 and 1 (Figure 2a,b), while Southern Africa and the Americas have a larger IBD peak around 0.5 (Figure 2a,b).

The genomic relationship matrix heatmap (Figure 2c) provides a visual summary of related accessions with individuals colour‐coded by geographic provenance in the columns and improvement status in the rows. As an example, a large group of accessions in the top left corner from Western Africa consisting of Advanced/Improved Cultivars, Breeding Lines, Landraces/Traditional Cultivars and Unknown accessions are similarly related. Another example is a small group of Landrace/Traditional Cultivars from Southern Africa in the bottom right corner that are highly related.

### Duplication within and between collections

Potential duplicate accessions among 10 617 cultivated cowpea were identified using Nei's genetic distance with a threshold of ≤0.03, Van Raden's genomic relatedness with a threshold of ≥1.3 and whole‐genome IBD with a threshold of ≥0.6. Each accession was compared with all other accessions in a pairwise manner for each of the metrics, resulting in a total of 112 710 072 pairwise combinations. Reciprocal pairs (e.g. Accession 1 versus Accession 2 and Accession 2 versus Accession 1) and identical pairs (e.g. Accession 1 versus Accession 1) were removed, leaving 56 355 036 unique combinations. Of these, 33 252 pairs met the three criteria and were classified as ‘potentially duplicated accessions’ (Table S3). A higher proportion of potential duplicates were detected within individual collections (57%) than between different collections (43%). Of these 33 252 pairs, 1329 were confirmed as ‘duplicated accessions’ based on passport alias names (Table S3). A higher proportion of duplicate accessions with matching names were detected between collections (74%) compared to within collections (26%). In cases where multiple pairs of accessions had an identical name, one or multiple accessions were retained depending on the genetic distance, GRM and IBD values. A total of 9609 non‐redundant accessions were retained for subsequent analyses to assess population structure.

### Global genetic structure of cowpea

#### Genetic structure

**Figure 3:**
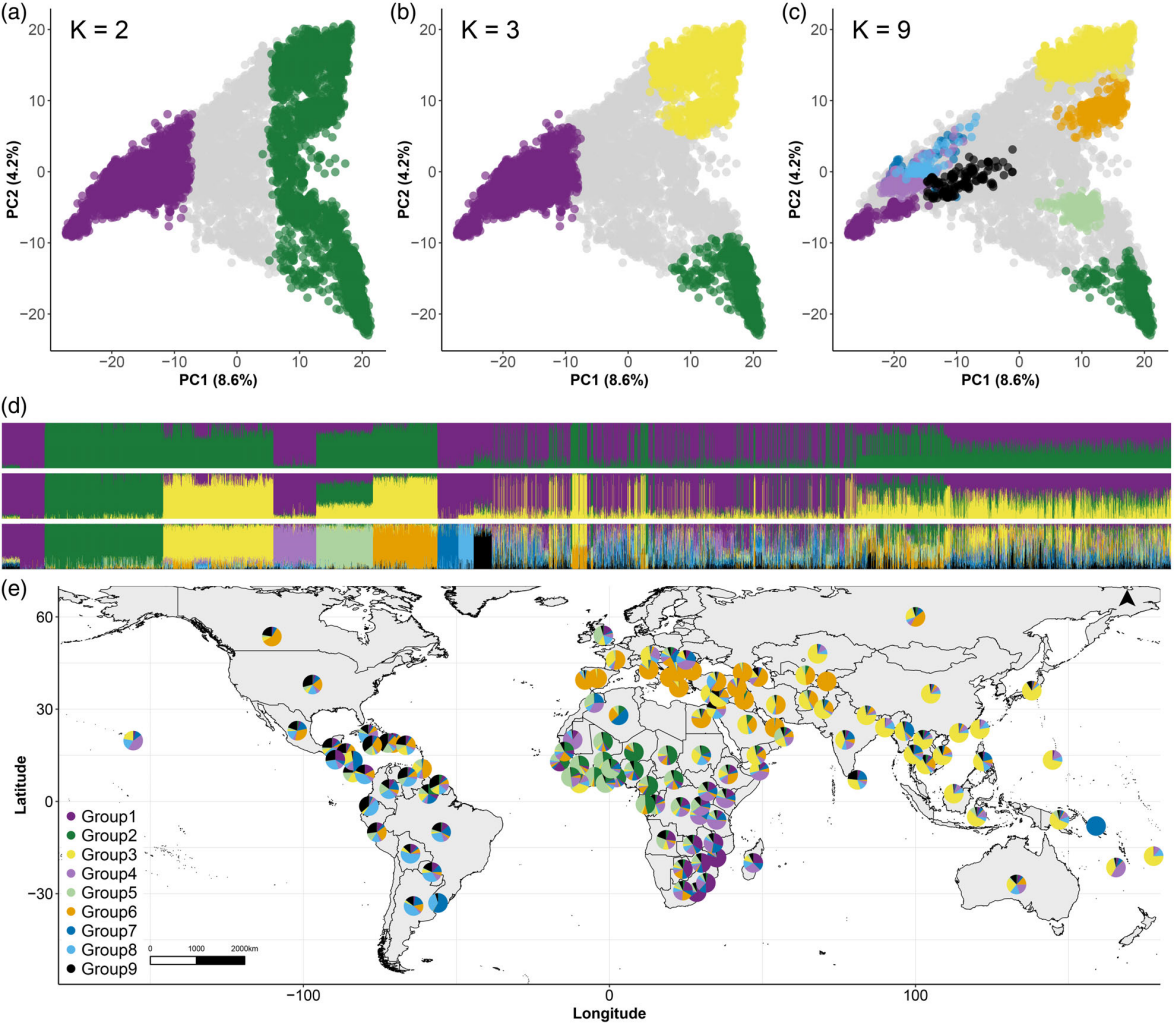
Population structure and geographic distribution of the non‐redundant global cowpea collection. (a–c) Principal component (PC) analysis of the 9609 accessions coloured by population at *K* = 2, *K* = 3 and *K* = 9. Colour key presented in (e). (d) Model‐based clustering of *K* = 3, *K* = 3 and *K* = 9. The *x*‐axis has accessions ordered according to their assignment to each *K* = 9 group. The *y*‐axes quantify the membership to each ancestral population represented in colours corresponding to each *K* = 9 group. Colour key presented in (e). (e) Geographic distribution of the genetic groups at *K* = 9. For each country, the pie represents the proportion of the ancestral allele frequencies.

The genetic population structure of 9609 cultivated cowpea accessions in the non‐redundant pruned dataset was inferred using three model‐based clustering algorithms implemented in ADMIXTURE, STRUCTURE and LEA (Frichot & François, 2015). Cross‐validation values from ADMIXTURE declined steadily with increasing *K*, without supporting a clear optimum, indicating the absence of a clearly defined optimal number of genetic groups in the dataset. However, the log‐likelihood and Δ*K* analyses indicated that *K* = 2 showed the best‐fit for both ADMIXTURE (Figure S3) and STRUCTURE (Figure S4). The sparse non‐negative matrix factorisation (sNMF) cross‐entropy analysis also supported two major populations, with further subdivision at higher *K* values (Figure S5). For all *K* values, an accession was assigned a genetic group if membership assignment to a group was greater than 70% (Table S4), while a lower percentage indicated admixture. At *K* = 2, the structure analysis separated the cowpea germplasm into Population 1 and Population 2. The ADMIXTURE, STRUCTURE and sNMF results at *K* = 2 were identical, so admixture proportions from sNMF are reported. The distribution of cowpea accessions between the two groups indicated that Population 2 (green) had the highest percentage of membership (43%) with 4115 accessions, while Population 1 (purple) had the lowest percentage of membership (35%) with 3415 accessions. However, the inferred ancestry indicated that 2079 accessions (22%) were admixed (Figure 3a,d, first panel). Support for *K* = 3 indicated further division of the accessions into three populations. Population 1 remained as Population 1 while Population 2 was divided into two Populations: Population 2 and Population 3 (Figure 3b,d, second panel). Population 1 (purple) had the highest percentage of membership (34%) with 3275 accessions, Population 3 (yellow) had an intermediate percentage of membership (20%) with 1959 accessions, and Population 2 (green) had the lowest percentage of membership (13%) with 1266 accessions. Splitting the accessions into three populations resulted in an increase in the number of accessions admixed between populations (*n* = 3109, 32%).

The populations at *K* = 2 and *K* = 3 reflect the broad genetic structuring in cowpea and have general geographic trends. At *K* = 2, accessions belonging to Population 1 originate from Western Africa, Eastern Africa, Southern Africa and the Americas. Accessions in Population 2 originate from Western and Central Africa, Europe/Mediterranean and Asia (Figure S6). At *K* = 3, when Population 2 was divided into Population 2 and Population 3, a clear distinction emerged, with African accessions in Population 2 and European/Mediterranean and Asian accessions in Population 3 (Figure S7).

We considered additional genetic structure to investigate the potential for population substructure across different regions. Support for a *K* = 9 model further divided the accessions into nine genetic groups (Figure S5). Population 1 split into Groups 1, 4, 7, 8 and 9; Population 2 split into Groups 2 and 5; and Population 3 split into Groups 3 and 6 (Figure 3c,d, third panel). Less than half of the 9609 accessions (*n* = 4025) were assigned to a group using a 70% membership threshold, with the remaining 5585 accessions admixed among groups (Figure S8).

#### Geographic enrichment of genetic groups

Geographic enrichment of the nine genetic groups was assessed using non‐parametric tests, focusing on group proportions rather than individual assignments. Five groups exhibited significant geographic separation: Group 2 in Africa, Group 3 in Asia and Oceania, Group 6 in Europe and Group 7 in South America (Table 1). The remaining groups (Groups 1, 4, 5, 8 and 9) did not reach statistical significance but showed the highest representation in specific regions: Group 1 in Southern Africa, Group 4 in Eastern Africa, Group 5 in Central and West Africa, Group 8 in South and Central America, and Group 9 in the Americas (North America, Central America, Southern America and the West Indies) (Table S5). To address regional sample size bias, each geographic region was rarefied to the same sample size (*n* = 50) and tested for significance over 500 permutations. Results from the rarefaction analysis were consistent with those obtained from the full dataset (Table S6). These results highlight both broad‐scale and region‐specific geographic patterns among cultivated cowpea, reflecting historical dispersal patterns.

**Table 1:** Non‐parametric assessment of geographic enrichment of group proportions for the nine genetic groups

Region	*n*	Group 1		Group 2		Group 3		Group 4		Group 5		Group 6		Group 7		Group 8		Group 9	
		Mean	Sig	Mean	Sig	Mean	Sig	Mean	Sig	Mean	Sig	Mean	Sig	Mean	Sig	Mean	Sig	Mean	Sig
Africa	5125	0.152	a	*0.272*	*a*	0.041	b	0.114	b	0.154	a	0.088	e	0.075	bc	0.045	e	0.059	c
Asia	2654	0.042	d	0.017	d	*0.429*	*a*	0.117	b	0.026	c	0.134	c	0.115	b	0.073	d	0.046	d
Central America	82	0.077	abc	0.009	bcd	0.103	b	0.084	bd	0.067	abc	0.122	bcde	0.076	bc	0.232	ab	0.230	ab
Europe	255	0.019	f	0.017	bc	0.107	bc	0.032	e	0.032	d	*0.678*	*a*	0.065	d	0.033	f	0.016	e
North America	968	0.039	e	0.017	c	0.060	c	0.066	c	0.064	b	0.222	b	0.096	c	0.151	c	0.284	a
Oceania	68	0.050	acd	0.018	bd	*0.322*	*a*	0.184	a	0.038	bcd	0.098	de	0.055	bc	0.143	bc	0.092	cd
South America	277	0.100	b	0.020	bd	0.044	b	0.078	ad	0.052	b	0.068	e	*0.156*	*a*	0.280	a	0.202	a
Unknown	180	0.053	cde	0.025	bc	0.133	b	0.096	bc	0.046	bc	0.195	bcd	0.102	bc	0.178	bc	0.173	b

*Note*: Mean group proportions for each region are presented with compact letter display indicating a significant difference in means across regions. Significant group enrichment for geographic regions are italicised. North and Central America are separated into two regions. *n* = Geographic region sample size.

#### Phylogeny of cowpea

**Figure 4:**
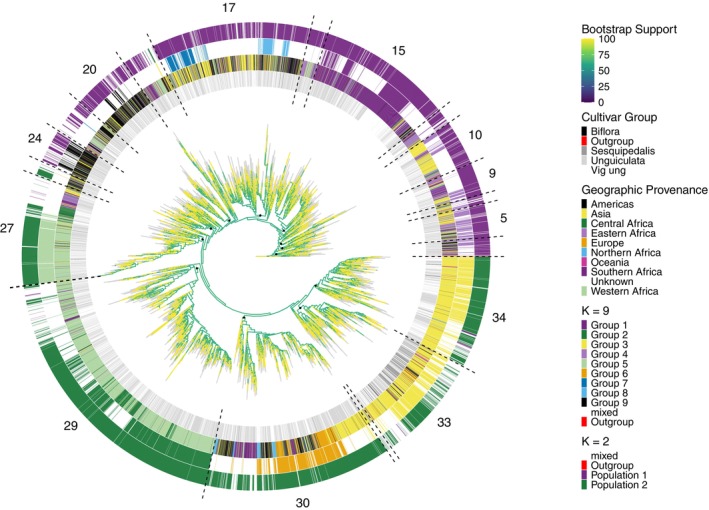
Maximum likelihood phylogeny of 9610 cowpea accessions implemented in IQ‐TREE. Bootstrap support values for each node are shown as a continuous colour scale on the branches from 0% in dark blue to 100% in yellow, while grey indicates the branches leading to the tips of the tree. Coloured bars on the outside of the tree ordered from the centre ring outwards show: the cultivar group, geographic provenance, genetic group at *K* = 9 and population at *K* = 2 for each accession. The 12 largest clades are numbered outside the tree with dashed lines indicating clade boundaries and black circles indicating their common ancestral node. See Table S7 for accession names and their corresponding clade.

The cowpea phylogenetic tree (Figure 4) is well supported, with all 9607 nodes showing bootstrap (BS) values >50% and a mean of 80.7% (Table S7). Support is highest for small terminal groups (BS 90–100%), whereas backbone edges are shorter and moderately supported (mean BS 64%). A total of 34 clades were identified along the backbone of the tree (Table S7), 12 of which contain over 200 accessions, including seven clades with more than 500 accessions. A subset of these large clades contains accessions that generally align with the populations, groups and have trends for improvement status, geographic provenance and cultivar group (Figure S9). A summary of the clades within this tree and detailed historic, taxonomic and geographic patterns can be found in Appendix S1, Figures S10– S17 and Table S7.

#### Genetic diversity and relationships between genetic groups

Analysis of molecular variance (amova) was computed between the populations and groups excluding the mixed accessions. Total molecular variance was partitioned into 32% between populations and 68% within populations at *K* = 2. At *K* = 3, total molecular variance was partitioned into 37% between populations and 63% within populations. At *K* = 9, total molecular variance was partitioned into 50% between groups and 50% within groups (Table 2). Although lower percentages of variation were found between groups at all hierarchy levels, all variance partitioning was statistically significant (*P* < 0.01). The analyses indicated high variance prevailing within the groups identified. amova was also computed between collections, and most molecular variation was partitioned within collections (95%) compared to between collections (5%; Table 2).

**Table 2:** Analysis of molecular variance (amova) partitioning the variance between and within genetic groups and collection

Hierarchy level	Source of variation	d.f.	SS	MS	*P*‐value	Variance (%)
	Between populations	1	975 285.8	975 285.8019	<0.001	31.95
Population (*K* = 2)	Within populations	7528	4 186 342.2	556.1028		68.05
	Total	7529	5 161 628	685.5662		100
	Between populations	2	1 198 696	599 347.781	<0.001	36.74
Population (*K* = 3)	Within populations	6497	3 338 295	513.821		63.26
	Total	6499	4 536 991	698.106		100
	Between groups	8	1 205 494	150 686.7596	<0.001	50.08
Group (*K* = 9)	Within group	4015	1 423 063	354.4365		49.92
	Total	4023	2 628 557	653.3822		100
	Between collections	6	200 120.5	33 353.4246	<0.001	5.32
Collection	Within collection	9602	6 346 552.6	660.9615		94.68
	Total	9608	6 546 673.2	681.3773		100

*Note*: *P*‐value calculated from 999 permutations.

Abbreviations: d.f., degrees of freedom; MS, mean square; SS, sum of squares.

**Figure 5:**
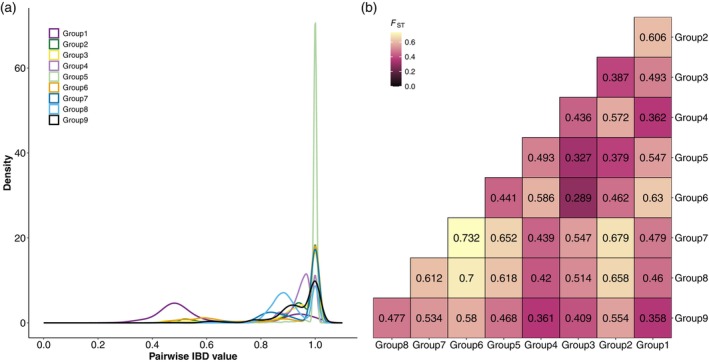
Population relatedness of the non‐redundant global cowpea collection. (a) Pairwise identity by descent (IBD) frequency distribution for each genetic group at *K* = 9. (b) Pairwise *F*_ST_ between genetic groups at *K* = 9, coloured continuously from low differentiation (black) to high differentiation (yellow).

Diversity metrics including the number of private alleles, allelic richness, nucleotide diversity, inbreeding coefficient (*F*_IS_), IBD, observed heterozygosity and the number of private alleles were assessed for each of the nine groups. Groups 1 (Southern Africa), 2 (Western/Central Africa) and 3 (Asia) contained the most private alleles (*n* = 1998–4641; Table S8) compared with Groups 4 (Eastern Africa), 5 (Central/Western Africa), 6 (Europe), 7 (South America), 8 (South/Central America) and 9 (Americas) (*n* = 0–656). Group 4 contained the most allelic richness (0.182) and nucleotide diversity (0.16) but a relatively high inbreeding coefficient (0.93) and few private alleles (*n* = 656; Table 3). Groups 1, 3 and 4 had large *π* values compared with Groups 7, 6 and 8. Groups 3–5 were the most inbred compared with Groups 1, 6–8 and Group 5 had the lowest observed heterozygosity (*H*_O_ = 0.007) (Table 3). Group 1 had the lowest inbreeding coefficient (*F*_IS_ = 0.77; Table 3), the largest observed heterozygosity (*H*_O_ = 0.033) and a large proportion of breeding material (i.e. $\hat{\pi }$ of approximately 0.5 is indicative of parent/child relationships and sib‐pairs; Figure 5a). Africa was the only continent to contain private alleles (Table S9) and had the largest nucleotide diversity and allelic richness (Table S10).

**Table 3:** Diversity metrics of the nine genetic groups

Group	Allelic richness	Private alleles	*π*	*F*_IS_	*H*_E_	*H*_O_
Group 1 (Purple)	1.72	2089	0.147	0.774	0.147	0.033
Group 2 (Green)	1.69	4641	0.104	0.907	0.104	0.010
Group 3 (Yellow)	1.74	1998	0.158	0.934	0.158	0.011
Group 4 (Light Purple)	1.82	656	0.161	0.932	0.160	0.011
Group 5 (Light Green)	1.60	630	0.108	0.937	0.108	0.007
Group 6 (Orange)	1.68	8	0.078	0.872	0.078	0.010
Group 7 (Blue)	1.73	0	0.065	0.846	0.065	0.010
Group 8 (Light Blue)	1.69	9	0.081	0.846	0.081	0.012
Group 9 (Black)	1.63	0	0.134	0.924	0.134	0.010

Abbreviations: *π*, weighted mean nucleotide diversity; *F*_IS_, weighted mean inbreeding coefficient; *H*_E_, expected heterozygosity; *H*_O_, observed heterozygosity.

Pairwise *F*_ST_, or the population stratification statistic, was estimated between the *K* = 9 groups (Figure 5b). The highest genetic differentiation of 0.73 was found between Group 6 (Mediterranean) and Group 7 (South American). Similar high differentiation of 0.7 was also observed between Group 6 and Group 8 (Central and South America). High pairwise *F*_ST_ values of 0.679 and 0.658 also indicated that Group 7 and Group 8 were distinct from Group 2 (West African). In contrast, the lowest genetic differentiation of 0.289 was found between Group 6 and Group 3 (Asian).

## DISCUSSION

The purpose of this study was to examine population structure and diversity of cultivated cowpea sourced from multiple genebanks and collections. The accessions in this collection reflect the global genetic diversity of extant cowpea. This dataset includes the largest cohort of cowpea genotyped using a single technology to date. The collection includes previously uncharacterised accessions and integrates previously characterised collections, creating a valuable resource for cowpea research and crop improvement.

### Geographic structure of genetic diversity mirrors historical dispersal

Cowpea was domesticated in sub‐Saharan Africa and subsequently spread across the Mediterranean Basin, Asian and the Americas (Herniter et al., 2020; Muñoz‐Amatriaín et al., 2021), creating multiple opportunities for founder effects, drift and selection under new agronomic and environmental conditions. As a result, the contemporary genetic structure of cowpea is expected to retain a geographic ‘footprint’ of historical dispersal routes and region‐specific improvement, an expectation supported by prior population genetic and genomic surveys of global and curated diversity sets (Fiscus et al., 2024; Huynh et al., 2013; Muñoz‐Amatriaín et al., 2021; Xiong et al., 2016). In particular, archaeological (D'andrea et al., 2007; Goldstein et al., 2024), textual (Herniter et al., 2020) and genetic evidence (Huynh et al., 2013; Xiong et al., 2016) suggests at least two major dispersal routes: one from Africa into the Old World (Mediterranean, India and Southeast Asia) (Fuller, 2003) and another into the New World during the Columbian Exchange (Herniter et al., 2020), consistent with the recurring observation that non‐African cowpea often clusters with distinct African regions (Fiscus et al., 2024; Herniter et al., 2020; Huynh et al., 2013; Muñoz‐Amatriaín et al., 2021; Xiong et al., 2016).

A substantial body of work supports West Africa as a major centre of diversity for cultivated cowpea and identifies broad geographic portioning among African gene pools, separating Western and Central African material and Eastern and Southern African material, and for non‐African accessions showing affinities to one or the other (Fatokun et al., 2018; Huynh et al., 2013; Padulosi & Ng, 1997; Steele, 1976). Huynh et al. (2013) identified two major African gene pools and showed that Asian and European accessions tend to share closer affinity with West African material, whereas many American accessions align more closely with East African material, suggesting a pattern consistent with historically distinct introduction routes and subsequent local diversification. Our results broadly agree with this framework: the two primary clusters detected in model‐based analyses are consistent with a deep, geographically structured partitioning of cowpea diversity, while finer structure reveals additional subdivisions that plausibly reflect multiple introductions, subsequent drift and selection during regional cultivation and breeding. This interpretation is also consistent with analyses of curated resources (Muñoz‐Amatriaín et al., 2021), which recover geographically patterned subpopulations and highlight the contribution of West African diversity to multiple lineages worldwide.

Interpreting the number of genetic clusters (*K*) requires explicit caution because STRUCTURE‐like models infer ancestry under assumptions (e.g. Hardy–Weinberg equilibrium within clusters, linkage equilibrium among loci) and require *post hoc* evaluation of *K*, which can be sensitive to both hierarchy and sampling design (Pritchard et al., 2000). The widely used delta *K* approach is known to preferentially identify the uppermost hierarchical level of structure and can show a bias towards *K* = 2 in many realistic scenarios, meaning that a ‘two‐population’ solution can represent an informative top‐level partition without capturing finer historical complexity (Janes et al., 2017) and should be interpreted as a broad summary rather than the full complexity of cowpea's global population history. Uneven sampling across regions and collections can further distort inference of *K* and ancestry proportions and has been highlighted as a limitation for STRUCTURE‐based interpretation unless accompanied by subsampling or sensitivity analyses. Accordingly, the additional structure inferred here using sNMF avoids assumptions of no genetic drift, Hardy–Weinberg equilibrium and linkage equilibrium in ancestral populations and is more appropriate to deal with inbred lineages (Frichot et al., 2014). Nevertheless, given the strong imbalance in sampling intensity among regions and collections, inferred groups and private‐allele patterns should be treated as indicative rather than definitive although rarefaction‐based geographic analyses were supportive of geographic enrichment of genetic groups (Table S6).

Phylogenetic reconstruction provides complimentary insight into relationships among accessions, but the short backbone and moderate support at deeper nodes observed here likely reflect a rapid radiation from Africa in which multiple lineages diverged over short time intervals, leaving limited phylogenetic signal for resolving basal relationships (Whitfield & Lockhart, 2007). In such contexts, incomplete lineage sorting and historical gene flow can generate substantial gene‐tree discordance (Degnan & Rosenberg, 2009; Hibbins & Hahn, 2021), and strictly bifurcating trees may inadequately represent evolutionary history (Bjornson et al., 2024), particularly in domesticated crops where introgression, and breeding create reticulate patterns (Degnan & Rosenberg, 2009; Hibbins & Hahn, 2021; Mallet, 2005). These processes complicate the reconstruction of deep evolutionary histories in rapid radiations, motivating the use of genome‐wide genetic datasets, coalescent–aware inference and network‐based representations that better accommodate admixture (Degnan & Rosenberg, 2009; Hibbins & Hahn, 2021; Liu et al., 2014; Philippe et al., 2011; Whitfield & Lockhart, 2007). Despite limited resolution at deep nodes, the cowpea phylogeny remains informative for more recent divergences, particularly among well‐supported smaller clades and clusters of closely related accessions, which are directly relevant for germplasm management (e.g. identifying clusters of closely related accessions) and for guiding the choice of diverse parents in breeding.

The strong relationship between inferred genetic groups and geographic origin indicates that spatial factors have significantly influenced the genetic differentiation among accessions and reinforces the value of geographically diverse sampling for both evolutionary inference and applied utilisation. From a conservation and breeding perspective, geographically structured diversity highlights reservoirs of alleles likely shaped by regional selection pressures and improvement histories, providing opportunities to prioritise underrepresented regions and guide the selection of accessions for breeding programmes aimed at improving crop performance under region‐specific constraints. To further resolve domestication history and intercontinental dispersal, future work integrating representative sampling of wild cowpea, plastome phylogenies and haplotype analysis of domestication‐related loci would provide stronger tests of single versus multiple domestication hypotheses and clarify the contribution of post‐domestication gene flow.

### Modern germplasm sharing and implications for cowpea conservation and management

Global sharing of cowpea germplasm has increased access to diverse genetic resources, but it also promotes redundancy through repeated collection and redistribution of identical or near‐identical material. In this global dataset, we identified 1008 duplicate accessions (9.5%), indicating that duplication is present but comparatively lower than reported in other crops, such as: barley, cassava and soybean which show 33% (Milner et al., 2019), 26% (Albuquerque et al., 2019) and 23% (Song et al., 2015) duplication, respectively. Differences in duplication rates among crops likely reflect a combination of collection history, naming or curation practices, and the resolution of genetic data used for detection, in addition to crop biology. Importantly, our estimate is likely conservative because the present study does not encompass all globally available accessions. Genesys currently holds approximately 45 500 amalgamated cowpea accessions (Data accessed through Genesys, https://www.genesys‐pgr.org/c/cowpea, 2 October 2025), and several major collections, such as the IITA (Ibadan, Nigeria, approximately 19 000 accessions), the NBPGR (New Delhi, India, approximately 3700 accessions) and the full collection held at the UCR (CA, USA, approximately 6000 accessions), contain substantial material not fully represented here. If these additional approximately 28 000 accessions were included in analyses, we would expect duplication within and among collections to increase, reinforcing the need for coordinated curation approaches that distinguish truly unique accessions from redundant entries.

Patterns of duplication provide insight into the processes generating redundancy. Accessions in this study originated from more than 120 countries, and re‐collecting from the same locations, combined with routine germplasm exchange between institutions, can readily produce duplicated entries across genebanks. Most duplicates were detected between collections (74%), indicating material exchange accompanied with appropriate documentation. However, the large number of molecularly similar pairs (33 252) highlights an additional challenge: high genetic similarity can reflect true duplication, but it can also reflect breeding relatedness (e.g. shared parentage, backcrossing to introduce simply inherited traits, or repeated use of elite lines in breeding programmes as noted by Boukar et al. (2020)). In practice, both processes likely contribute. This distinction matters operationally, because redundancy inflates curation costs and can lead to users unknowingly select duplicates if accessions are sampled randomly. While closely related breeding materials may remain valuable, appropriate pedigree, passport and standardised trait metadata will greatly enhance and support downstream use.

Limitations in passport metadata further complicate both management and biological interpretation. Only 4113 accessions in this global collection were geo‐referenced or resolved beyond country level (Table S1), constraining fine‐scale geographic inference and reducing our ability to link genetic patterns to specific environments. However, this limitation can be partially mitigated by integrating genetic data with a Focused Identification of Germplasm Strategy (FIGS) (Sunitha et al., 2024). By linking poorly annotated accessions to genetically similar accessions (Tables S3 and S11) with high‐quality collection site information, FIGS provides a practical path to infer likely adaptive traits and prioritise subsets of germplasm for evaluation (Khazaei et al., 2013). This integrative approach would enable breeders and users to move beyond broad or random screening towards more targeted identification of candidate accessions likely to contain adaptive or agronomic traits aligned with specific breeding objectives.

From a management perspective, this study suggests that substantial molecular variance is retained within collections, rather than between (Table 2), indicating that local collections contain distinct germplasm, supporting continued investment in regional collections and emphasising the value of coordinated global curation. Given declining sequencing and genotyping costs, a feasible long‐term goal is a globally coordinated genotyping‐based curation effort across genebanks to identify unique accessions, flag candidate duplicates and standardise identity tracking across genebanks (Anglin et al., 2025). In this context, this study demonstrates that low‐density SNP markers can serve as an efficient initial screening tool to identify candidate duplicate sets for curator review. These candidates can then be prioritised for confirmatory evaluation using higher density genotyping and/or phenotypic evaluation (Song et al., 2015). Importantly, new field collections should be genotyped and assessed for similarity to pre‐existing accessions to prevent accumulated redundancy and support strategic decisions about which accessions should be prioritised for additional back up of irreplaceable accessions.

IBD analyses further supports the interpretation that both true duplication and pedigree‐related relationships contribute to the observed patterns. The strong IBD peak around 1 is consistent with many similar and near‐identical accessions (Figure 2a,b), while the IBD peak around 0.5 in regions such as Southern Africa and the Americas (Figure 2a,b) suggests large contributions of breeding material (parent/offspring and half‐sib relationships) originating from these regions. Several limitations should be acknowledged. First, within‐accession heterogeneity was not assessed; although cowpea is predominantly selfing with low outcrossing rates of 0–5% (Fatokun & Ng, 2007; Gumede et al., 2022; Xiong et al., 2016), within‐accession heterogeneity could still influence pairwise similarity and duplicate detection in some cases. Second, duplication inference is constrained by marker density: while the 3073 SNPs used here are sufficient for broad screening, structure and diversity estimates, they may fail to resolve very closely related material from non‐identical material. Therefore, the candidate duplicate list should be treated as a prioritised set for germplasm management follow‐up rather than a definitive catalogue. Corroborating evidence for shared origin can be sought through (1) comparison of vouchers in the genebanks' herbarium, (2) inspection of the original (paper) passport records, (3) assessing higher density marker data for a larger number of individuals from candidate duplicates and (4) field evaluation in which candidate duplicates are grown side by side. Although these steps are labour intensive, the upfront investment is likely to be outweighed by the long‐term cost of maintaining superfluous material and the downstream inefficiencies created by redundant sampling.

Taken together, these findings emphasise that effective cowpea germplasm conservation now requires both genetic curation and standardised information management (Milner et al., 2019). Improving the consistency of passport descriptors and standardising trait and pedigree data, supported by ongoing digital efforts across genebanks, will significantly enhance the accuracy, accessibility and long‐term value of germplasm collections. In parallel, genotype‐informed curation provides a framework for reducing redundancy, prioritising globally unique accessions for long‐term conservation and safety duplication, and enabling breeders to more efficiently identify germplasm with likely adaptive potential.

## MATERIALS AND METHODS

### Plant materials

A total of 10 617 cultivated cowpea (*V. unguiculata*) accessions and one sister subspecies accession (*V. unguiculata* subsp. *stenophylla*) were used in this study. These accessions originated from diverse geographic regions and seven collections. The majority of these accessions were from the USDA Genebank (63%; *n* = 6735), followed by the IITA Genebank, Nigeria (18%; *n* = 1942), representing the core collection of cowpea accessions described by Mahalakshmi et al. (2007). A collection of accessions held at AGG, Australia (7%; *n* = 791) and the NARO Genebank, Japan (4%; *n* = 414), were included to expand geographic distribution. The diversity of the cowpea accessions was further enriched by the inclusion of several smaller but highly valuable collections from various regions. These included 351 accessions from the UCR mini‐core collection (Muñoz‐Amatriaín et al., 2021), 271 accessions from the National Genebank of Mozambique at the IIAM (Macharia et al., 2025) and 114 accessions from the Central and Southern American collection from Unidad de Genómica Avanzada, LAN. Together, these diverse sources represent a broad spectrum of genetic variation, including both regional and global cowpea germplasm, thereby providing a comprehensive foundation for subsequent genetic studies. Passport descriptors (i.e. metadata) including taxonomic classification, location of provenance, collection site and improvement status were collated for all accessions from their respective collection, where available. Metadata were accessed online through: the USDA‐ARS Germplasm Resources Information Network (GRIN) (https://www.ars‐grin.gov/), IITA Genebank (https://my.iita.org/accession2/browse.jspx), AGG GRIN‐Global Database System (https://ausgenebank.agriculture.vic.gov.au/gringlobal/search), NARO (https://www.gene.affrc.go.jp/databases‐plant_search_en.php), Genesys (https://www.genesys‐pgr.org/) and Supporting Information from Muñoz‐Amatriaín et al. (2021) and Macharia et al. (2025). Metadata for accessions used are provided in Table S1.

### Genotyping‐by‐sequencing

Genotyping of the global cowpea collection was conducted by Diversity Arrays Technology (DArT, Canberra, ACT, Australia) Pty Ltd (https://www.diversityarrays.com/services/dartseq/) using the DArTseq™ reduced‐representation sequencing platform (Kilian et al., 2012). For each accession, 30–40 mg of seed tissue derived from single or multiple seeds was sampled and placed in a 96‐well plate. Samples were ground in a TissueLyser II (QIAGEN, Hilden, Germany) using metal rods at 30 Hz for 20–30 sec, repeated twice. Following grinding, the rods were removed and tubes sealed. Ground samples were stored at 4°C before shipping to DArT where DNA extraction was conducted using a modified cetyl trimethyl ammonium bromide (CTAB) method (Doyle & Doyle, 1987). For accessions restricted by quarantine regulations, leaf material from the NARO accessions were sampled following the procedure described by Ofem et al. (2025) and sent to DArT for DNA extraction. Samples from the IIAM collection were extracted at the Genebank in Mozambique using the protocol described by Macharia et al. (2025) and subsequently shipped to DArT for sequencing. All DNA samples were then digested with methylation sensitive restriction enzymes (*Pst*I and *Mse*I) to reduce genome complexity and to remove repetitive sequences (Kilian et al., 2012). Libraries with size lengths of 200–500 bp were constructed using a TruseqNano DNA HT Sample Preparation Kit (Illumina; catalogue no. FC‐121‐4003) following the manufacturer's recommendations and sequenced on an Illumina HiSeq 2500 platform to produce 77 bp paired end reads (Sansaloni et al., 2011). Raw sequencing reads were processed using DArT's proprietary analytical pipeline. In this pipeline, the FASTQ files were filtered to remove adapters, low‐quality reads and redundant reads with barcode regions stringently filtered (Phred pass score ≥30) to ensure accurate sample assignment to the correct reads. Identical reads were collapsed into ‘fastqcall’ tag files which were then used as input for DArT's secondary pipeline for SNP analysis (Raman et al., 2014). Reads were subsequently aligned to the cowpea reference genome IT97K‐499‐35 v1.2 (Lonardi et al., 2019) using BWA‐MEM v0.7.17 (Li & Durbin, 2009), and SNP genotypes were called with DArT's proprietary secondary pipeline (DArTsoft14) (Sansaloni et al., 2020). Genotype data were delivered in VCF format (Cruz et al., 2013). During post‐DArT quality control, samples with a call rate ≤0.50 and markers with a call rate ≤0.80 were excluded. The overall call rate in the raw genotypic dataset was approximately 82%. Missing genotypes were inferred using Beagle v 5.0 (Browning & Browning, 2016). Further filtering was applied by removing markers with heterozygosity ≥0.4 and individuals with heterozygosity ≥0.2. The final filtered dataset consisted of 10 618 accessions and 4290 high‐quality SNPs, denoted the ‘raw dataset’, providing a robust dataset for subsequent analyses of genetic diversity. The genomic distribution and density of these SNPs were visualised using the online SNP Density tool (https://www.bioinformatics.com.cn/en) (Figure S1).

### Genetic relatedness between collections and dataset generation

Cultivated cowpea were extracted from the raw dataset and markers with a minor allele frequency ≥0.01 were retained using VCFtools v 0.1.16 (Danecek et al., 2011), resulting in 3073 SNPs across 10 617 accessions denoted the ‘cultivated dataset’. An initial principal component analysis (PCA) was performed using the *prcomp* function from the stats package in R v 4.3.0 (R Core Team, 2023) to visualise accession distribution according to their metadata, including cultivar group, improvement status, collection (i.e. germplasm source) and geographic provenance.

Duplicate accessions within the cultivated dataset were identified based on three metrics: genetic distance, genomic relatedness and IBD. Pairwise Nei's genetic distance was calculated using the *nei.dist* function from the poppr package v 2.9.4 (Kamvar et al., 2014), ranging from 0 (minimal genetic difference) to 1 (maximal genetic difference) using the cultivated dataset. A conservative threshold of ≤0.03 was used to identify genetically similar pairs. Van Raden's genomic relationship matrix was calculated using the *Gmatrix* function from the AGHmatrix package v 2.1.4 (Amadeu et al., 2023) using the cultivated dataset, with pairwise values ≥1.3 considered indicative of genetic relatedness. Linkage disequilibrium (LD) pruning was performed using PLINK v 1.90b7.2 (Purcell et al., 2007) on the cultivated dataset with the parameter *‐‐indep‐pairwise 50‐5‐0.5* to generate the ‘cultivated‐pruned dataset’, consisting of 2302 unlinked SNPs. This dataset was used to calculate identify by state (IBS), defined as the average proportion of alleles shared between pairs of individuals at genotyped SNPs. The proportion of whole‐genome IBD was also calculated using the *‐‐genome* flag and PLINK's Hidden Markov Models (HMM) algorithm (Purcell et al., 2007). Accessions with a $\hat{\pi }$ value greater than 0.5 indicated parent/child relationships, sib‐pairs and duplicated accessions. Accession pairs would need to fit all three criteria to be considered a potential duplicate: genetic distance ≥0.03, genomic relatedness ≥1.3 and $\hat{\pi }$ ≥0.6. To adopt a more conservative approach, passport metadata were also considered: accession pairs that met all three genetic criteria were required to share the same accession name or alias. Potentially duplicate accessions with shared names were thus identified as duplicates and removed from the raw dataset using bcftools v 1.15.1 (Danecek et al., 2021). This dataset was filtered to remove markers with a minor allele frequency ≤0.01 resulting in 3083 SNPs and 9609 accessions and was denoted the ‘non‐redundant dataset’. The non‐redundant dataset was also LD pruned using the methods described above, resulting in 2312 SNP retained to generate the ‘non‐redundant pruned dataset’.

In the cultivated‐pruned dataset, relatedness was summarised as the proportion of accession pairs with $\hat{\pi }$ >0 within and between collections, reported as ‘per cent pairwise IBD’ in Figure 1b. Relatedness was calculated for all pairs between 10 617 accessions using PLINK v 1.90b7.2 (Purcell et al., 2007) by summing the pairs with $\hat{\pi }$ >0 within a collection combination, then calculating the total number of pairs within the collection combination and finally calculating the percentage of related pairs relative to the total possible combinations for the collection combination. In addition, the distribution of $\hat{\pi }$ values within each geographic provenance was assessed with density plots in R, reported as ‘pairwise IBD values’ in Figure 2a,b.

### Genetic structure and diversity analysis

To explore the overall patterns of genetic clustering in cowpea germplasm, two separate analyses were performed: (1) Model‐based structure analyses used ADMIXTURE v 1.3.0 (Alexander et al., 2009), STRUCTURE v 2.3.4 (Pritchard et al., 2000) and the sNMF algorithm (Frichot et al., 2014) implemented in the LEA package v 3.12.2 (Frichot & François, 2015); and (2) Phylogeny reconstruction used IQ‐TREE v 2.2.2.3 (Minh et al., 2020). The ‘non‐redundant pruned dataset’ described above was used for structure analyses and phylogenetic analyses.

ADMIXTURE, STRUCTURE and sNMF were used to perform model‐based clustering analyses to explore the broad patterns of population structure and determine the number of putative ancestral populations (*K*). STRUCTURE uses a Bayesian clustering approach, ADMIXTURE uses maximum likelihood modelling, and sNMF uses sparse nonnegative matrix factorization algorithms to estimate individual ancestry coefficients. As ADMIXTURE is computationally faster to run compared with STRUCTURE, the ADMIXTURE analysis was performed using 10 independent runs specified with a different seed using ‐s with each run set from 1 to 40 with 1‐ cross‐validations (‐‐cv = 10) to determine the *K* values with the lowest cross‐validation error. As the cross‐validation error continued to decline as *K* increased, the optimal *K* value was determined using the delta *K* method (Evanno et al., 2005). Subsequently, 10 independent runs for each simulated value of *K* ranging from 1 to 15 was performed using STRUCTURE. Each run consisted of a burn‐in period of 50 000 iterations, followed by 100 000 Markov Chain Monte Carlo (MCMC) replications. The putative optimal *K* was determined using the pophelperShiny app v 2.1.1 (Francis, 2017) using the delta *K* method (Evanno et al., 2005). In parallel, model‐based estimation of ancestral coefficients was performed using the sNMF algorithm (Frichot et al., 2014) implemented in the R package LEA (Frichot & François, 2015). The number of ancestral populations (*K*) was set from 1 to 25 with 20 iterations for each *K* value. The best‐fit *K* value was determined by assessing the change in cross‐entropy for each *K*, that is the elbow of the curve. Ancestral proportions from the sNMF analysis were plotted using the PophelperShiny app v 2.1.1 (Francis, 2017). Accessions were assigned to populations if their individual admixture coefficient matrix (*Q*) was at least 70%, and those with less than 70% membership probability were considered mixed. Population structure was further examined using the *prcomp* function to perform a PCA. Distribution of genetic groups to country of provenance was visualised in R using the rworldmap v 1.3‐8 (South, 2011) and ggplot2 v 3.5.1 packages (Wickham, 2016).

For the phylogeny reconstruction, one *V. unguiculata* subsp. *stenophylla* accession was included as the outgroup in the non‐redundant pruned dataset. A maximum likelihood phylogeny was generated using IQ‐TREE v 2.2.2.3 (Minh et al., 2020) specifying the polymorphisms‐aware phylogenetic model (Schrempf et al., 2016, 2019). ModelFinder (Kalyaanamoorthy et al., 2017) was first used to determine the best‐fit substitution model for the data (GTR+F+ASC+R10). Default parameters were used for tree construction with the *stenophylla* accession assigned as the outgroup. Convergence was not assessed by undertaking independent runs of IQ‐TREE and examining tree topology due to the large number of tips (9610) and nodes, but did ensure that the log‐likelihood values were stable at the end of the run. Branch support was performed using 10 000 replicates of UFboot (Hoang et al., 2018). The 9610 taxa tree was plotted in R using ggtree v 3.8.2 (Yu et al., 2017) and ggtreeExtra v 1.10.0 (Xu et al., 2021).

Relatedness in terms of the proportion of IBD was calculated for all pairwise accessions using the ‘non‐redundant pruned dataset’ and the distribution of these proportions were assessed within each group using a density plot in R. To assess genetic diversity, the non‐redundant dataset was used to estimate six diversity metrics. Nucleotide diversity (*π*; Hohenlohe et al., 2010), *F*_IS_ (Weir & Cockerham, 1984), pairwise *F*_ST_ (Weir & Cockerham, 1984), expected and observed heterozygosity (*H*_E_ and *H*_O_, respectively) were calculated for each of the genetic groups and eight geographic regions using snpR v 1.2.9.2 (Hemstrom & Jones, 2023). Allelic richness (Hurlbert, 1971) and the number of private alleles for groups and regions were calculated using hierfstat v 0.5‐11 (Goudet, 2005) and poppr v 2.9.4 (Kamvar et al., 2014, 2015), respectively. amova was used to partition the total variance in allele frequencies within and among hierarchies to assess the significance level at each partition: *K* = 2, *K* = 3, *K* = 9 and collection, and was implemented in the packages poppr v2.9.4 (Kamvar et al., 2014, 2015) and ade4 v1.7‐22 (Dray & Dufour, 2007).

### Geographic enrichment assessment of genetic groups

Collection metadata was used to derive geographic origin for each accession, with a small number from an unknown origin (*n* = 180). Geographic enrichment of each genetic group was assessed using individual ancestry proportions (*Q*‐values) as continuous response variables. For each group, differences in *Q*‐value distributions among geographic regions were tested using non‐parametric Kruskal–Wallis rank‐sum tests and, when significant, pairwise Wilcoxon rank‐sum tests with a Bonferroni correction were applied to evaluate significant enrichment based on geography. To account for sample bias, we rarefied each region to 50 accessions without replacement across 500 replicates and tested for enrichment using the methodology mentioned above. After assessing broad‐scale geographic enrichment, fine‐scale geographic regions were assessed using the same two tests using the *kruskal.test* and *pairwise.wilcox.test* from the stats package in R 4.3.0 (R Core Team, 2023). Regions with fewer than 10 accessions, including the British Isles, Micronesia, Polynesia and Western Europe, were removed prior to analysis.

## AUTHOR CONTRIBUTIONS

EM, DJ, AC and AK designed the project. PM, MMW, AC, MD'A, TI, JC, PO‐A, J‐PV‐C and SN collected and provided the plant materials. TS prepared the sequenced samples. SP, AH and SS performed data analyses. SP wrote the manuscript. EM, DJ, MD'A, MMW, SS, AH, AK, PO‐A, YT, JC, TI and SN revised the manuscript.

## CONFLICT OF INTEREST

The authors declare no competing interests.

## Supporting information


**Appendix S1.** Summary of the cowpea phylogenetic tree and geographic relationships.

**Figure S1.** Heatmap showing the density and distribution of 4290 SNP markers across the 11 cowpea chromosomes.

**Figure S2.** Principal component (PC) analysis of 10 617 accessions with each panel representing various metadata.

**Figure S3.** Support for the number of ancestral populations (*K*) from the ADMIXTURE analysis where *K* was tested from 1 to 40.

**Figure S4.** Probability support for the number of ancestral populations (*K*) from the STRUCTURE analysis where *K* was tested from 1 to 25.

**Figure S5.** Cross‐entropy support for the number of ancestral populations (*K*) from the sNMF analysis where *K* was tested from 1 to 25.

**Figure S6.** Geographic distribution of the Populations at *K* = 2.

**Figure S7.** Geographic distribution of the Populations at *K* = 3.

**Figure S8.** Principal component (PC) analysis of the 9609 cowpea accessions coloured by group (*K* = 9).

**Figure S9.** Maximum likelihood phylogeny of 9610 cowpea accessions implemented in IQ‐TREE.

**Figure S10.** Maximum likelihood phylogeny of 894 cowpea accessions from Clade 15.

**Figure S11.** Maximum likelihood phylogeny of 1056 cowpea accessions from Clade 17.

**Figure S12.** Maximum likelihood phylogeny of 384 cowpea accessions from Clade 20.

**Figure S13.** Maximum likelihood phylogeny of 733 cowpea accessions from Clade 27.

**Figure S14.** Maximum likelihood phylogeny of 1874 cowpea accessions from Clade 29.

**Figure S15.** Maximum likelihood phylogeny of 1241 cowpea accessions from Clade 30.

**Figure S16.** Maximum likelihood phylogeny of 712 cowpea accessions from Clade 33.

**Figure S17.** Maximum likelihood phylogeny of 748 cowpea accessions from Clade 34.

**Table S1.** Passport descriptors of 10 618 cowpea accessions.
**Table S2.** Percentage of improvement status category assignment for 10 617 cultivated cowpea accessions for each collection source.
**Table S3.** Paired potential duplicate accessions (33 252) and paired duplicate accessions (1329) identified from genetic distance (GD), genomic relatedness (GRM), whole‐genome identity by descent (PI HAT, Z0, Z1 and Z2), and accession name and alias matching.
**Table S4.** Admixture proportion assignment of each accession to three values of *K*.
**Table S5.** Non‐parametric assessment of regional geographic enrichment of group proportions for nine genetic groups.
**Table S6.** Non‐parametric assessment of regional geographic enrichment of group proportions for nine genetic groups using rarefaction without replacement with 50 samples per region and 500 permutations.
**Table S7.** Tree data for the circular 9610 cowpea tree, including node, branch lengths, bootstrap support and accession order.
**Table S8.** Counts of private alleles within each genetic group at *K* = 9.
**Table S9.** Counts of private alleles within each continent.
**Table S10.** Diversity metrics for each continent.
**Table S11.** Predicted group membership of 1008 redundant cowpea accessions at three levels of *K*.
**Table S12.** Number of accessions within each clade by their population assignment at *K* = 2.
**Table S13.** Number of accessions within each clade by their population assignment at *K* = 3.
**Table S14.** Number of accessions within each clade by their group assignment at *K* = 9.
**Table S15.** Percentage of accessions within each clade by their geographic provenance.
**Table S16.** Percentage of accessions within each clade by their improvement status.
**Table S17.** Percentage of accessions within each clade by their collection source.
**Table S18.** Percentage of accessions within each clade by their cultivar group.
**Table S19.** Regional geographic locations for American (Northern, Central and Southern) accessions and their corresponding clade.

## Data Availability

The data that support the findings of this study are available at UQ eSpace in the dataset ‘CowpeaDiversityGeneticData’ using the following link: https://doi.org/10.48610/659a65b, in the Supporting Information of this article, and in the following GitHub repository: https://github.com/SofiePearson/CowpeaDiversity. Genomic data are currently being integrated and can be accessed through an interactive web application called AGG Pretzel (https://agg.plantinformatics.io/).
