# Supplementary material for: Scaling up orphan crop research: genebank genetics highlight geographic structure in cultivated cowpea from 10 617 global accessions
Source: Plant J. 2026 Mar 14;125(6):e70777. doi: 10.1111/tpj.70777 (PMC12988651; doi:10.1111/tpj.70777)
Supplement: Supplementary file 2 — Figure S1. Heatmap showing the density and distribution of 4290 SNP markers across the 11 cowpea chromosomes. [file TPJ-125-0-s006.pdf]

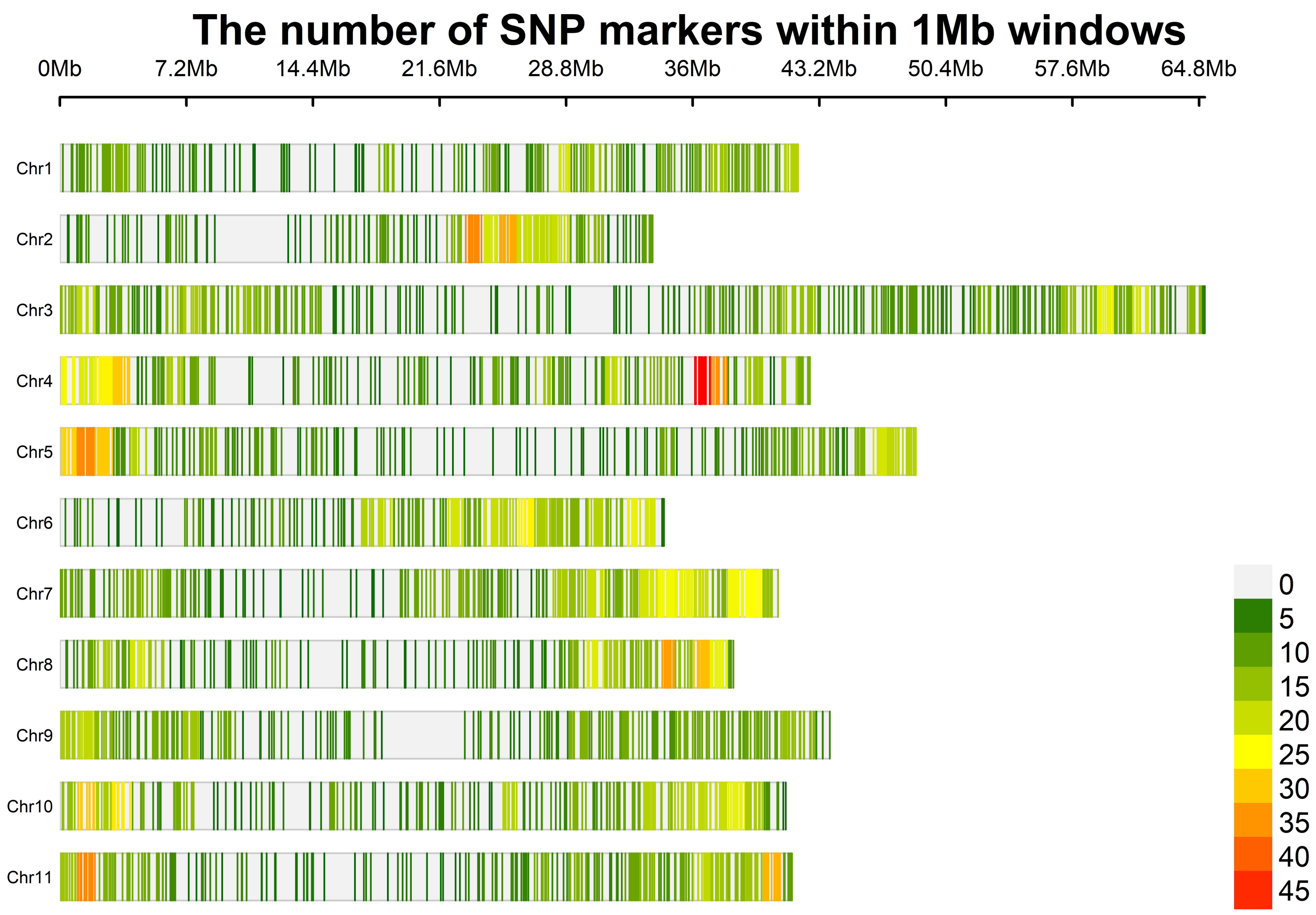

Figure S1. Heatmap showing the density and distribution of 4,290 SNP markers across the eleven cowpea chromosomes.
