# Supplementary material for: Scaling up orphan crop research: genebank genetics highlight geographic structure in cultivated cowpea from 10 617 global accessions
Source: Plant J. 2026 Mar 14;125(6):e70777. doi: 10.1111/tpj.70777 (PMC12988651; doi:10.1111/tpj.70777)
Supplement: Supplementary file 4 — Figure S3. Support for the number of ancestral populations (K) from the ADMIXTURE analysis where K was tested from 1 to 40. [file TPJ-125-0-s009.pdf]

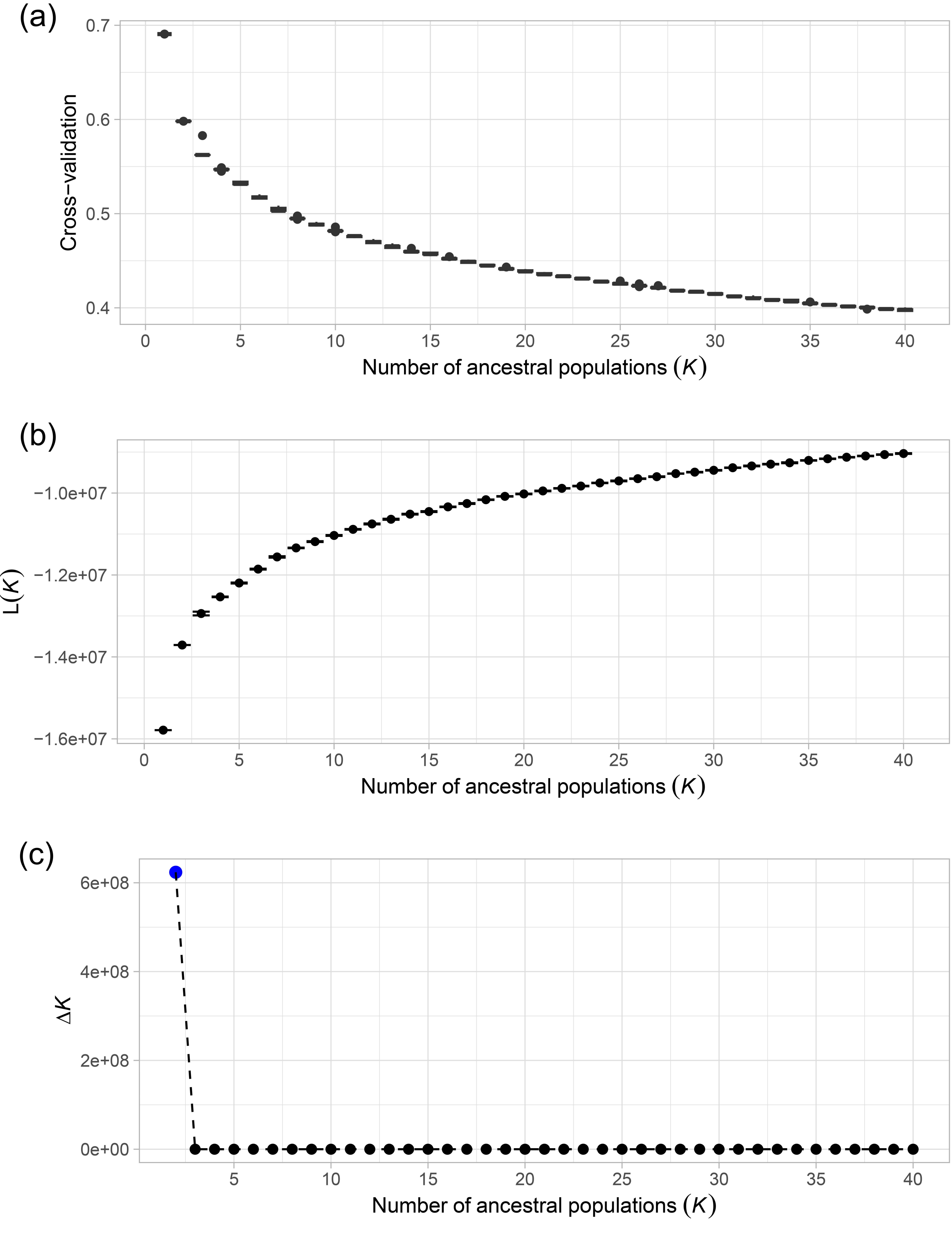

Figure S3. Support for the number of ancestral populations (*K*) from the ADMIXTURE analysis where *K* was tested from 1 to 40.
(a) Cross-validation values and standard error from 10 independent runs for *K* = 1 to *K* = 40.
(b) Loglikelihood values and standard error for *K* = 1 to *K* = 40.
(c) Delta *K* for *K* = 2 to *K* = 40. The *K* = 2 value with the greatest support is highlighted in blue.
