# Supplementary material for: Scaling up orphan crop research: genebank genetics highlight geographic structure in cultivated cowpea from 10 617 global accessions
Source: Plant J. 2026 Mar 14;125(6):e70777. doi: 10.1111/tpj.70777 (PMC12988651; doi:10.1111/tpj.70777)
Supplement: Supplementary file 5 — Figure S4. Probability support for the number of ancestral populations (K) from the STRUCTURE analysis where K was tested from 1 to 25. [file TPJ-125-0-s012.pdf]

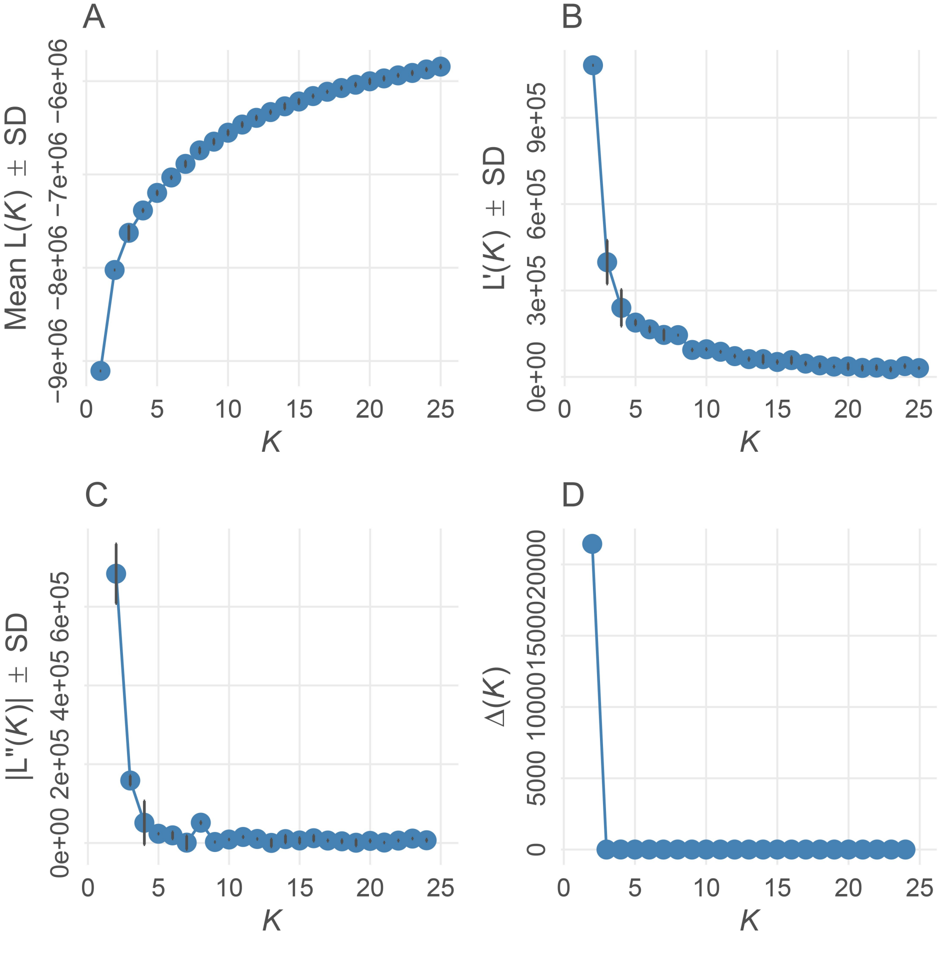

Figure S4. Probability support for the number of ancestral populations (*K*) from the STRUCTURE analysis where *K* was tested from 1 to 25. Plots produced from the Evanno method.
