# Supplementary material for: Scaling up orphan crop research: genebank genetics highlight geographic structure in cultivated cowpea from 10 617 global accessions
Source: Plant J. 2026 Mar 14;125(6):e70777. doi: 10.1111/tpj.70777 (PMC12988651; doi:10.1111/tpj.70777)
Supplement: Supplementary file 6 — Figure S5. Cross‐entropy support for the number of ancestral populations (K) from the sNMF analysis where K was tested from 1 to 25. [file TPJ-125-0-s014.pdf]

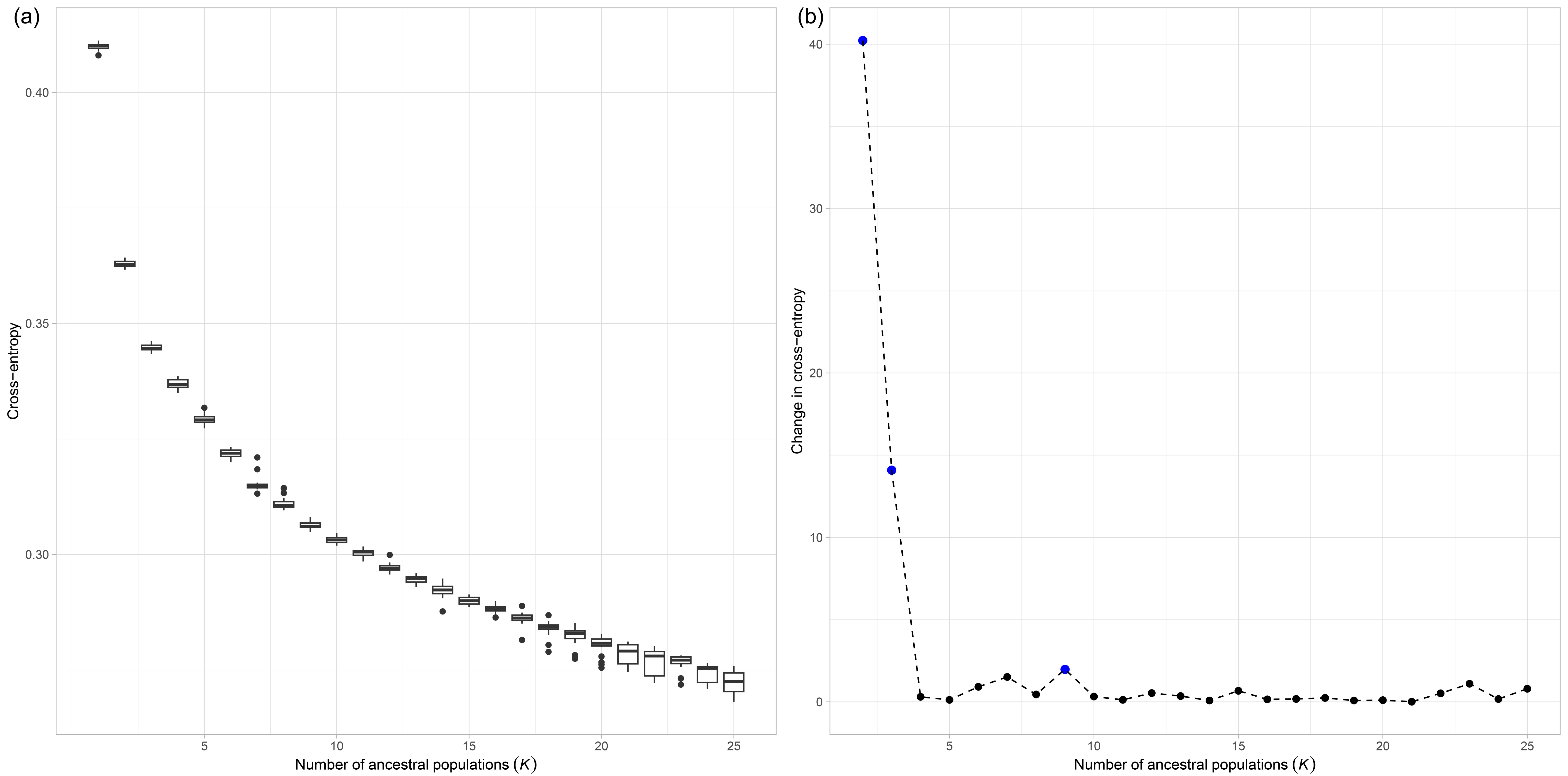

Figure S5. Cross-entropy support for the number of ancestral populations (*K*) from the sNMF analysis where *K* was tested from 1 to 25.
(a) Cross-entropy boxplots from 20 iterations for *K* values from 1 – 25.
(b) Change in cross-entropy values with *K* = 2, *K* = 3 and *K* = 9 highlighted in blue.
