# Supplementary material for: Scaling up orphan crop research: genebank genetics highlight geographic structure in cultivated cowpea from 10 617 global accessions
Source: Plant J. 2026 Mar 14;125(6):e70777. doi: 10.1111/tpj.70777 (PMC12988651; doi:10.1111/tpj.70777)
Supplement: Supplementary file 7 — Figure S6. Geographic distribution of the Populations at K = 2. [file TPJ-125-0-s003.pdf]

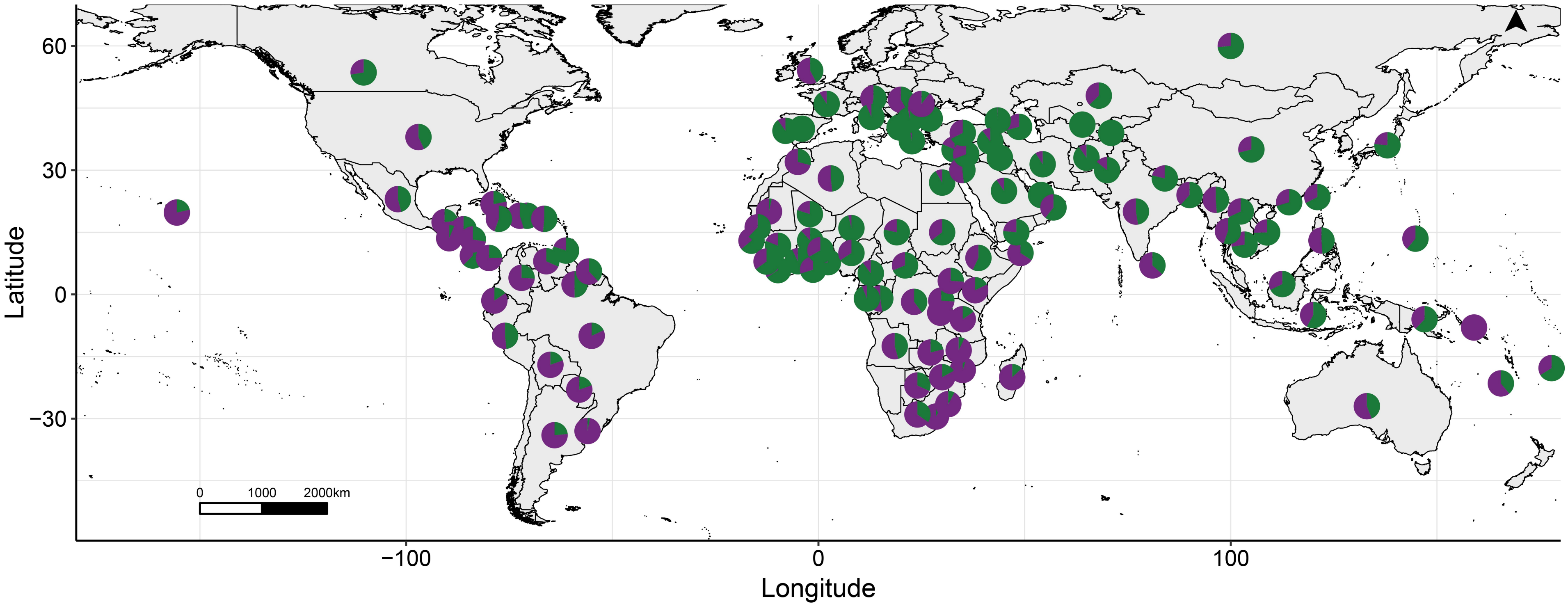

Figure S6. Geographic distribution of the Populations at *K* = 2. For each country, the pie represents the proportion of the ancestral allele frequencies. Purple = Population 1 and Green = Population 2.
