# Supplementary material for: Scaling up orphan crop research: genebank genetics highlight geographic structure in cultivated cowpea from 10 617 global accessions
Source: Plant J. 2026 Mar 14;125(6):e70777. doi: 10.1111/tpj.70777 (PMC12988651; doi:10.1111/tpj.70777)
Supplement: Supplementary file 9 — Figure S8. Principal component (PC) analysis of the 9609 cowpea accessions coloured by group (K = 9). [file TPJ-125-0-s013.pdf]

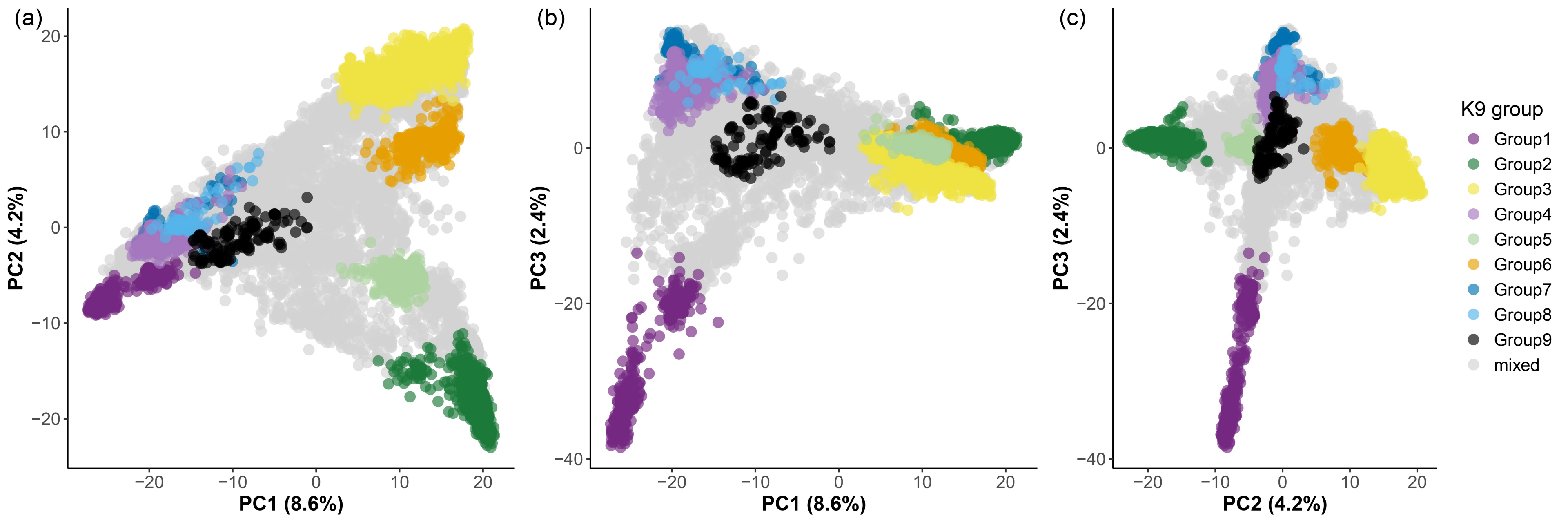

Figure S8. Principal component (PC) analysis of the 9,609 cowpea accessions coloured by group (*K* = 9).
(a) PC1 vs PC2.
(b) PC1 vs PC3.
(c) PC2 vs PC3.
