# Supplementary figures and images for: Scaling up orphan crop research: genebank genetics highlight geographic structure in cultivated cowpea from 10 617 global accessions
Source: Plant J. 2026 Mar 14;125(6):e70777. doi: 10.1111/tpj.70777 (PMC12988651; doi:10.1111/tpj.70777)

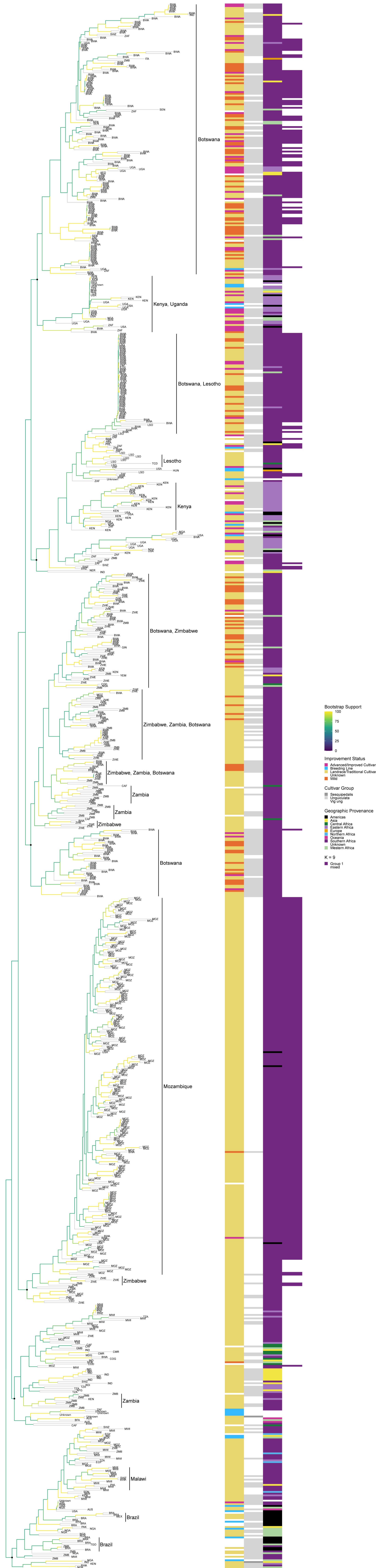

Supplement: Supplementary file 11 — Figure S10. Maximum likelihood phylogeny of 894 cowpea accessions from Clade 15. [file TPJ-125-0-s008.pdf]

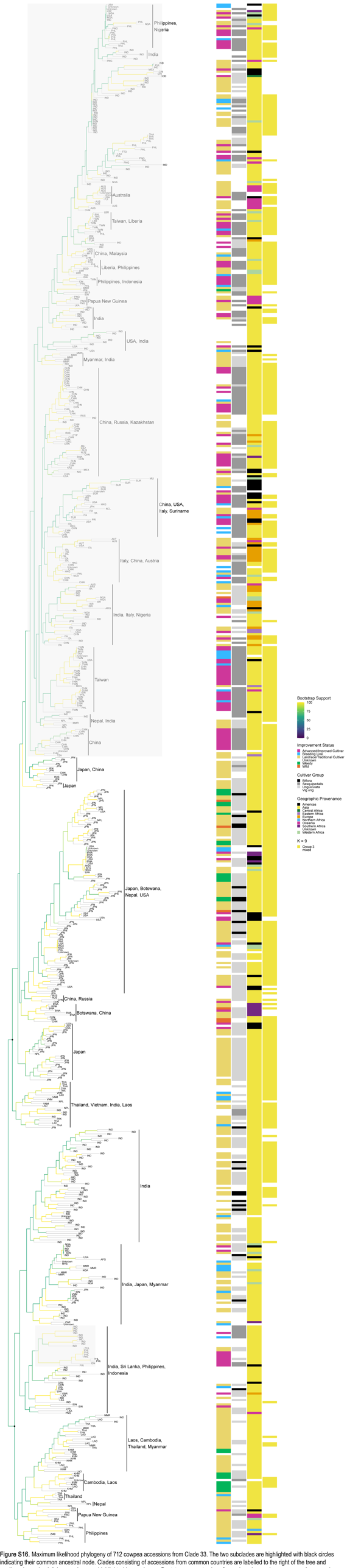

Supplement: Supplementary file 17 — Figure S16. Maximum likelihood phylogeny of 712 cowpea accessions from Clade 33. [file TPJ-125-0-s005.pdf]

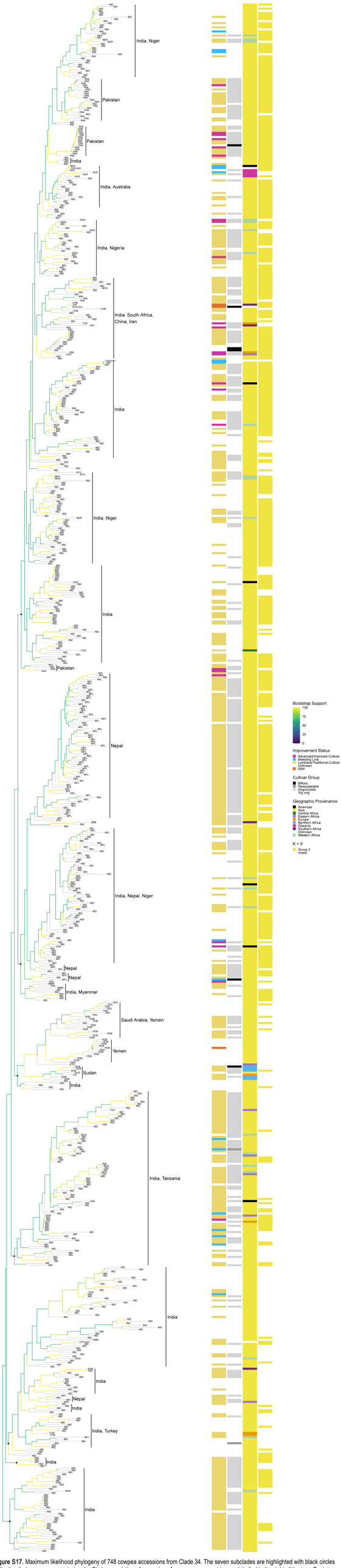

Supplement: Supplementary file 18 — Figure S17. Maximum likelihood phylogeny of 748 cowpea accessions from Clade 34. [file TPJ-125-0-s010.pdf]
