# Supplementary material for: Scaling up orphan crop research: genebank genetics highlight geographic structure in cultivated cowpea from 10 617 global accessions
Source: Plant J. 2026 Mar 14;125(6):e70777. doi: 10.1111/tpj.70777 (PMC12988651; doi:10.1111/tpj.70777)
Supplement: Supplementary file 16 — Figure S15. Maximum likelihood phylogeny of 1241 cowpea accessions from Clade 30. [file TPJ-125-0-s018.pdf]

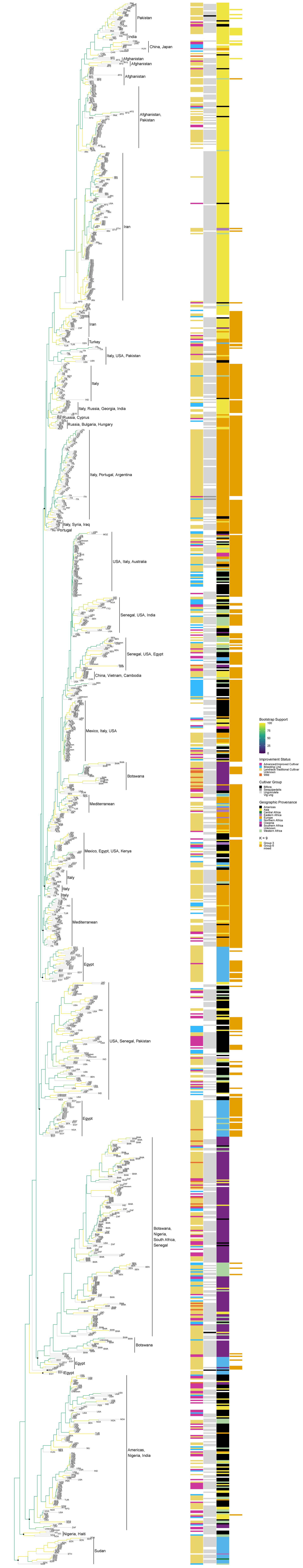

Figure S15. Maximum likelihood phylogeny of 1,241 cowpea accessions from Clade 30. The 11 subclades are highlighted with black circles indicating their common ancestral node. Clades consisting of accessions from common countries or geographic regions are labelled to the right of the tree. Bootstrap support values for each node are shown as a continuous colour scale on the branches from 0% in dark blue to 100% in yellow, while grey indicates the branches leading to the tips of the tree. Three letter country code of the country of provenance is presented for each accession at the tip. Coloured bars on the right-hand side of the tree, ordered from the left to the right show: the improvement status, cultivar group, geographic provenance, and genetic group at K = 9 for each accession. Colour keys are presented to the right of the bars. See **Table S7** for accession names and their corresponding subclade and order in the tree.
